# Production of extracellular reactive oxygen species by phytoplankton: past and future directions

**DOI:** 10.1093/plankt/fby039

**Published:** 2018-09-26

**Authors:** Julia M Diaz, Sydney Plummer

**Affiliations:** Department of Marine Sciences, Skidaway Institute of Oceanography, University of Georgia, Savannah, GA, USA

**Keywords:** harmful algae, phytoplankton bloom, oxidative stress, redox homeostasis, biological interactions, monitoring, cryptic biogeochemistry

## Abstract

In aquatic environments, phytoplankton represent a major source of reactive oxygen species (ROS) such as superoxide and hydrogen peroxide. Many phytoplankton taxa also produce extracellular ROS under optimal growth conditions in culture. However, the physiological purpose of extracellular ROS production by phytoplankton and its wider significance to ecosystem-scale trophic interactions and biogeochemistry remain unclear. Here, we review the rates, taxonomic diversity, subcellular mechanisms and functions of extracellular superoxide and hydrogen peroxide production by phytoplankton with a view towards future research directions. Model eukaryotic phytoplankton and cyanobacteria produce extracellular superoxide and hydrogen peroxide at cell-normalized rates that span several orders of magnitude, both within and between taxa. The potential ecophysiological roles of extracellular ROS production are versatile and appear to be shared among diverse phytoplankton species, including ichthyotoxicity, allelopathy, growth promotion, and iron acquisition. Whereas extracellular hydrogen peroxide likely arises from a combination of intracellular and cell surface production mechanisms, extracellular superoxide is predominantly generated by specialized systems for transplasma membrane electron transport. Future insights into the molecular-level basis of extracellular ROS production, combined with existing high-sensitivity geochemical techniques for the direct quantification of ROS dynamics, will help unveil the ecophysiological and biogeochemical significance of phytoplankton-derived ROS in natural aquatic systems.

## INTRODUCTION

Reactive oxygen species (ROS) include intermediates in the four-electron reduction of oxygen to water: superoxide, hydrogen peroxide and hydroxyl radical. Biological ROS production has been the subject of scientific inquiry since the discovery of the ubiquitous antioxidant enzyme, superoxide dismutase (SOD) (McCord and Fridovich, 1969). Since then, it has been well established that all oxygen-metabolizing organisms generate ROS and that this ROS production has potentially self-harmful effects. Yet more recently, awareness has been growing that biological ROS production can promote growth and survival. Extracellular ROS production regulates cell differentiation by fungi (Aguirre *et al.*, 2005), innate immunity in seaweeds (Weinberger, 2007) and white blood cells (Babior, 1999), heterotrophic feeding by corals (Armoza-Zvuloni *et al.*, 2016) and reproduction by sea urchins (Shapiro, 1991). Extracellular ROS production by the harmful bloom-forming phytoplankton species *Chattonella marina* has been implicated in its toxicity (Kim and Oda, 2010), growth (Oda *et al.*, 1995) and iron acquisition (Garg *et al.*, 2007; Liu *et al.*, 2007), while many other phytoplankton generate extracellular ROS under non-stressful conditions for reasons that remain mysterious.

ROS occur naturally in the environment, as the products of both abiotic and biologically driven chemical reactions. In natural waters, ROS are present at low concentrations (10^−18^−10^−6^ mol L^−1^) and are short-lived (μsec–days), yet ubiquitous (Table I). Aquatic ROS profoundly shape the biogeochemical cycling of carbon, as well as toxic and nutrient metals (Siciliano *et al.*, 2002; Pullin *et al.*, 2004; Barbeau, 2006; Learman *et al.*, 2011; Rose, 2012; Zinser, 2018). In oxygenated surface waters, biological production of ROS can be a substantial and dominant ROS source, especially in areas of elevated biological productivity, such as phytoplankton blooms (Rose *et al.*, 2008b, 2010; Hansard *et al*., 2010; Vermilyea *et al.*, 2010; Rusak *et al.*, 2011; Dixon *et al.*, 2013; Marsico *et al.*, 2015; Cory *et al.*, 2016). Despite prodigious extracellular ROS production by many cultivated phytoplankton species and the quantitative contribution of phytoplankton communities to aquatic ROS fluxes, the physiological significance of phytoplankton-derived ROS and the wider implications for food web dynamics and biogeochemistry are poorly understood. Here, we review the rates, mechanisms and ecophysiological roles of extracellular ROS production by phytoplankton, with a focus on marine taxa and the ROS superoxide and hydrogen peroxide. We also propose future research directions to clarify the ecosystem-scale significance of phytoplankton-derived ROS.

**Table I:** Typical concentrations and lifetimes of ROS in aquatic systems

ROS		Concentration (mol L^−1^)	Lifetime
Superoxide	O_2_−	10^−12^–10^−9^	sec–min
Hydrogen peroxide	H_2_O_2_	10^−9^–10^−6^	hours–days
Hydroxyl radical	OH•	10^−18^–10^−15^	μsec

## SURVEY OF EXTRACELLULAR ROS PRODUCTION BY PHYTOPLANKTON

**Fig. 1.**
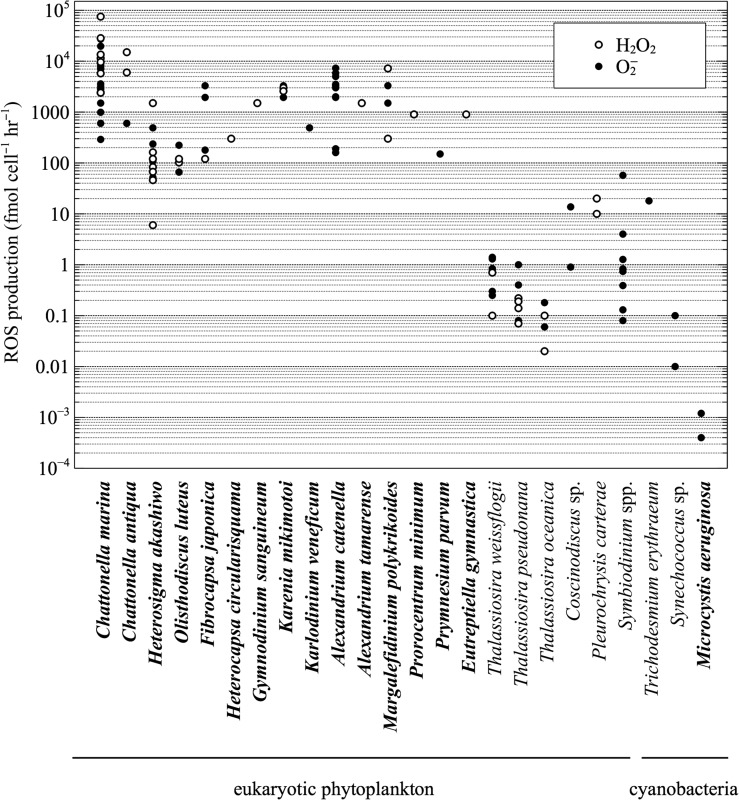
Survey of extracellular superoxide (O_2_−) and hydrogen peroxide (H_2_O_2_) production rates by phytoplankton. Species known to form HABs are indicated in bold. Data and original references are provided in Supplementary Table S1.

A broad diversity of phytoplankton produce extracellular ROS under optimal growth conditions in culture, including eukaryotic phytoplankton and cyanobacteria (Fig. 1; Supplementary Table S1). The major ROS superoxide (Oda *et al.*, 1997; Marshall *et al.*, 2005a; Portune *et al.*, 2010; Mooney *et al.*, 2011; Dorantes-Aranda *et al.*, 2015; Schneider *et al.*, 2016; Cho *et al.*, 2017), hydrogen peroxide (Oda *et al.*, 1997; Kim *et al.*, 1999a; Portune *et al.*, 2010; Schneider *et al.*, 2016; Cho *et al.*, 2017) and hydroxyl radical (Oda *et al.*, 1992a; Yang *et al.*, 1995; Cho *et al.*, 2016) have all been examined in phytoplankton. Compared to previous studies on hydroxyl radical production, however, the literature on superoxide and hydrogen peroxide generation by phytoplankton is much more expansive. Thus, the focus here is primarily on superoxide and hydrogen peroxide.

Extracellular ROS production has been quantified on a per-cell basis in at least 21 eukaryotic phytoplankton species, and the majority of these are capable of forming harmful algal blooms (HABs) (Yang *et al.*, 1995; Kim *et al.*, 1999a; Marshall *et al.*, 2005a; Mooney *et al.*, 2011; Cho *et al.*, 2017). Raphidophytes are the most thoroughly studied HAB group in terms of the production and potential functions of extracellular ROS, especially the *Chattonella* species *C. marina* and *C. antiqua* (Oda *et al.*, 1997; Marshall *et al.*, 2003, 2005a, 2005b), as well as *Heterosigma akashiwo*, *Olisthodiscus luteus* and *Fibrocapsa japonica* (Oda *et al*., 1992b; Yang *et al.*, 1995; Oda *et al.*,^1997^; Marshall *et al.*, 2005a; Portune *et al.*, 2010). Extracellular ROS production has been examined in harmful bloom-forming dinoflagellates, including *Alexandrium* spp. (Marshall *et al.*, 2005a; Mooney *et al.*, 2011; Dorantes-Aranda *et al.*, 2015; Mardones *et al.*, 2015; Cho *et al.*, 2017), *Margalefidinium polykrikoides* (Kim *et al.*, 1999a, 2002; Tang and Gobler, 2009b; Griffith and Gobler, 2016) and *Karenia mikimotoi* (Yamasaki *et al.*, 2004; Dorantes-Aranda *et al.*, 2015; Cho *et al.*, 2017). Among non-HAB forming eukaryotic phytoplankton, extracellular ROS are produced by the symbiotic dinoflagellates *Symbiodinium* spp. (Saragosti *et al.*, 2010; Zhang *et al.*, 2016a), the coccolithophorid *Pleurochrysis carterae* (Palenik *et al.*, 1987) and diatoms, including the genus *Thalassiosira* (Kustka *et al.*, 2005; Rose *et al.*, 2008b; Milne *et al.*, 2009; Waring *et al.*, 2010; Schneider *et al.*, 2016). Extracellular superoxide production has also been quantified in at least four species of cyanobacteria (Rose *et al.*, 2005, 2008b; Godrant *et al.*, 2009; Fujii *et al.*, 2011; Hansel *et al.*, 2016).

Cell-normalized rates of extracellular ROS production vary widely among phytoplankton species (10^−4^–10^5^ fmol cell^−1^ h^−1^; Fig. 1;Supplementary Table S1). The ichthyotoxic raphidophyte *C. marina* is capable of the highest extracellular ROS production rates, yet other HAB species can reach similar rates of ROS production, including *Alexandrium catenella* (Mardones *et al.*, 2015), *M. polykrikoides* (Kim *et al.*, 1999a, 2002), *K. mikimotoi* (Yamasaki *et al.*, 2004; Dorantes-Aranda *et al.*, 2015; Cho *et al.*, 2017) and *F. japonica* (Dorantes-Aranda *et al.*, 2015). Overall, HAB-forming eukaryotes generate much more extracellular ROS than other phytoplankton taxa, including the harmful freshwater cyanobacterium *Microcystis aeruginosa* (Fujii *et al.*, 2011), as well as non-HAB species such as *Thalassiosira* spp. Cell size is a major control on the interspecific variability in extracellular ROS production (Oda *et al.*, 1997; Marshall *et al.*, 2005a; Diaz *et al.*, 2013), yet when accounting for cell size, some harmful algae still generate substantially more ROS than non-harmful species (Kustka *et al.*, 2005). Thus, to some degree, extracellular ROS production may be related to the tendency of some phytoplankton species to form HABs.

In addition to large interspecific variability, extracellular ROS production rates also vary considerably within phytoplankton species (<10 to 10^3^-fold; Fig. 1; Supplementary Table S1). Major factors underlying this variability include growth phase (Kawano *et al.*, 1996; Kim *et al.*, 1999a, 2004; Skeen *et al.*, 2004; Garg *et al.*, 2007; Portune *et al.*, 2010), cell density (Yang *et al.*, 1995; Twiner and Trick, 2000; Kim *et al.*, 2002; Marshall *et al.*, 2005b; Dorantes-Aranda *et al.*, 2015), cell lysis (Dorantes-Aranda *et al.*, 2013; Mardones *et al.*, 2015), inter-strain differences (Ishimatsu *et al.*, 1996; Oda *et al.*, 1997; Portune *et al.*, 2010; Dorantes-Aranda *et al.*, 2013; Mardones *et al.*, 2015), irradiance (Kim *et al.*, 1999a; Dorantes-Aranda *et al.*, 2013), temperature (Twiner and Trick, 2000), salinity (Mardones *et al.*, 2015) and iron availability (Twiner and Trick, 2000). Examining the effect of these parameters on extracellular ROS production by phytoplankton has helped to illuminate potential ecophysiological functions (see section Ecophysiological roles of phytoplankton-derived extracellular ROS).

## SUBCELLULAR PATHWAYS OF EXTRACELLULAR ROS PRODUCTION

**Fig. 2.**
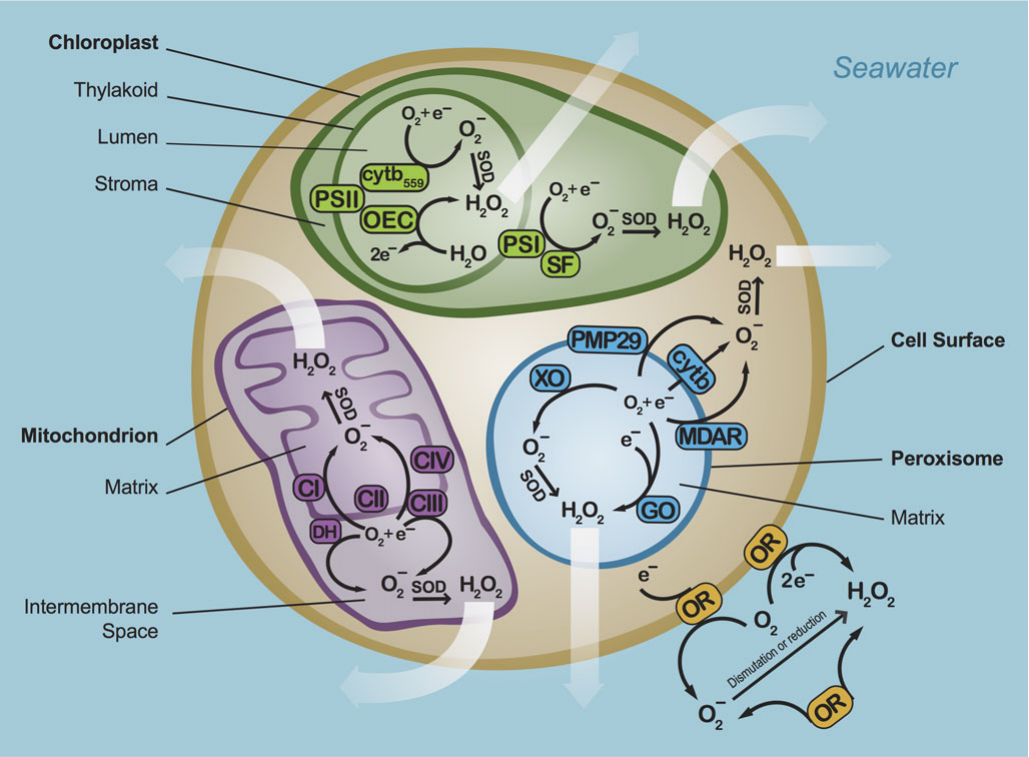
Mechanisms of superoxide (O_2_−) and hydrogen peroxide (H_2_O_2_) production in phytoplankton. Chloroplast: PSI—photosystem I, PSII—photosystem II, SF—stromal factor, OEC—oxygen-evolving complex, cytb_559_—cytochrome b559; Mitochondria: CI—complex I, CIII—complex III, DH—NAD(P)H dehydrogenase; Peroxisomes: GO—glycolate oxidase, XO—xanthine oxidase, MDAR—monodehydroascorbate reductase, cytb—cytochrome b, PMP29—peroxisome membrane polypeptide 29; Cell surface and cell-free environment: OR—oxidoreductase. Intracellular hydrogen peroxide can diffuse within and outside of cells (white arrows), but intracellular superoxide is unlikely to escape the cell.

In phytoplankton, ROS production occurs at several major sites: the chloroplasts and mitochondria (or thylakoid membranes in cyanobacteria), peroxisome (eukaryotes only), cell surface and the cell-free environment (Fig. 2). The principal ROS-generating reaction at these sites is the formation of superoxide by the single-electron reduction of oxygen. In turn, rapid dismutation of superoxide by SOD is a primary mechanism for the production of hydrogen peroxide. Hydrogen peroxide can also be produced independently from superoxide through the two-electron reduction or oxidation of oxygen and water, respectively (Fig. 2).

Intracellular ROS arise through several key processes. For example, in chloroplasts, ROS production within the stromal fluid occurs via the Mehler reaction, which is mediated by photosystem I (PSI; probably via iron-sulfur clusters) and/or with the involvement of a stromal factor (SF) such as monodehydroascorbate reductase (MDAR) (Asada, 1999, 2006). ROS production also occurs in the chloroplast lumen at several sites within photosystem II (PSII) such as the oxygen-evolving complex (OEC) and cytochrome b559 (cytb_559_), although ROS production in PSII is thought to be minor compared to PSI (Pospíšil, 2014). In mitochondria, intracellular production of ROS in the matrix and intermembrane space is primarily mediated by complex I (CI), complex III (CIII) and membrane-bound NAD(P)H dehydrogenases (DH) (Møller, 2001; Murphy, 2009). ROS production within the peroxisome matrix occurs via several oxidoreductases such as glycolate oxidase (GO) and xanthine oxidase (XO). On the peroxisome membrane, ROS are produced via NAD(P)H-dependent reactions mediated by MDAR, cytochrome b (cytb), and/or peroxisome membrane polypeptide 29 (PMP29) (Noctor *et al.*, 2002; Foyer *et al.*, 2009; del Rio and Lopez-Huertas, 2016).

The movement of superoxide and hydrogen peroxide within the intracellular space differs strongly, such that fundamentally different processes are likely involved in the biogenic fluxes of these ROS into the environment. For example, intracellular hydrogen peroxide readily diffuses across membranes, which may be an important route for the release of biogenic hydrogen peroxide into seawater, as seen for *C. marina* (Kim *et al.*, 2000a, 2007). However, as a much shorter-lived (~μs) anion at physiological pH with a limited diffusive distance (~100 s of nm), superoxide does not readily cross biological membranes (Korshunov and Imlay, 2002; Lesser, 2006). Even the complete lysis of cells under severe oxidative stress cannot release enough superoxide to account for the steady-state concentrations that have been measured in natural waters (Rose, 2012). Intracellular processes such as photosynthesis are, therefore, unlikely to be a direct source of biologically derived extracellular superoxide. Indeed, *Symbiodinium* spp. (Saragosti *et al.*, 2010; Zhang *et al.*, 2016a) and *Thalassiosira* spp. (Schneider *et al.*, 2016) produce extracellular superoxide in the dark, indicating the presence of non-photosynthetic mechanisms for superoxide production. Furthermore, the photosynthetic inhibitor dichlorophenyldimethylurea (DCMU) does not alter extracellular superoxide production by *C. marina* and *H. akashiwo* (Oda *et al.*, 1998).

Rather than originating from intracellular sources, most extracellular superoxide is likely produced directly at the cell surface. Cell surface NADPH oxidoreductases catalyze the production of extracellular superoxide in many organisms, including protozoa, seaweeds, fungi, plants, animals (Saran, 2003; Aguirre *et al.*, 2005; Hervé *et al.*, 2005; Bedard *et al.*, 2007; Weinberger, 2007; Anderson *et al.*, 2011) and the freshwater alga *Chlamydomonas reinhardtii* (Anderson *et al.*, 2016). In fact, extracellular superoxide production by C. *marina* occurs on the cell surface through an NADPH oxidoreductase that is homologous to human neutrophil NADPH oxidase (Shimada *et al.*, 1993; Kim *et al.*, 2007). Light exposure stimulates extracellular superoxide production by *C. marina* (Dorantes-Aranda *et al.*, 2013; Li *et al.*, 2015), *Thalassiosira* spp. (Schneider *et al.*, 2016) and *Symbiodinium* spp. (Saragosti *et al.*, 2010), which suggests that photosynthesis (Kim *et al.*, 1999a; Marshall *et al.*, 2002) may play an indirect role in extracellular superoxide production by supplying NADPH to the cell surface NADPH oxidoreductase, as suggested previously (Saragosti *et al.*, 2010). In addition to superoxide, extracellular hydrogen peroxide may also be directly generated at the cell surface. For example, extracellular hydrogen peroxide production by *Prymnesium parvum* is mediated by amino acid oxidases during metabolism of exogenous organic nitrogen sources (Palenik *et al.*, 1988).

Extracellular ROS production can also occur in the cell-free environment. For instance, the *C. marina* NADPH oxidoreductase can become dislodged from the cell surface and actively generate superoxide in cell-free spent media (Kim *et al.*, 2000a, 2007). Cell-free hydrogen peroxide production has also been documented for the model diatom *Phaeodactylum tricornutum* (Schneider *et al.*, 2016), although the mechanism remains unresolved.

## ECOPHYSIOLOGICAL ROLES OF PHYTOPLANKTON-DERIVED EXTRACELLULAR ROS

ROS commonly arise as metabolic byproducts, whose damaging effects on vital biomolecules such as DNA, lipids and proteins are well known (Lesser, 2006). However, ROS production can be directed through specialized pathways (Fig. 2) to participate in a variety of regulatory and signaling processes that aid in the growth and survival of the organism making the ROS (Foyer and Noctor, 2005; Lesser, 2006; Mittler *et al.*, 2011; Scheibe and Dietz, 2012; Schmitt *et al.*, 2014). As discussed below, extracellular ROS production by phytoplankton may modulate biological interactions such as HAB toxicity, allelopathy, grazing and viral infection, while also aiding in growth and iron acquisition. ROS may serve many of these functions in the same phytoplankton species while also sharing similar purposes across different phytoplankton taxa. For instance, even though *Chattonella* is the most prolific producer of extracellular ROS among phytoplankton, the role of extracellular ROS in this genus may not be unique, as outlined in the following sections.

### Ichthyotoxicity of HABs

ROS-forming HABs have caused immense financial losses to aquaculture industries in Australia (Hallegraeff *et al.*, 1998), Japan (Okaichi, 1997) and Chile (Fuentes *et al.*, 2006; Mardones *et al.*, 2010). ROS are involved in the noxious or toxic effects of several HAB-forming species, such as raphidophytes (Yang *et al.*, 1995; Oda *et al.*, 1997; Kim *et al.*, 1999b), and the dinoflagellates *M. polykrikoides* (Kim *et al.*, 1999a; Tang and Gobler, 2009b, 2010) and *Alexandrium* spp. (Flores *et al.*, 2012; Mardones *et al.*, 2015). For example, antioxidants alleviate the toxic effect of multiple HAB species on various marine organisms (Yang *et al.*, 1995; Oda *et al.*, 1992b, 1997; Kim *et al.*, 1999a, 1999b; Tang and Gobler, 2009a, 2009b, 2010; Flores *et al.*, 2012). Furthermore, fish mucus and/or surface receptor-binding lectins stimulate ROS production by several raphidophytes, suggesting a role for extracellular ROS in modulating these interactions (Tanaka *et al.*, 1994; Nakamura *et al.*, 1998; Oda *et al.*, 1998; Kim *et al.*, 1999b, 2000b; Kim and Oda, 2010).

Although ROS may be involved in some cases of HAB toxicity, the mechanism(s) are still controversial. For example, *C. marina* is thought to cause fish death by inducing suffocation via gill tissue damage, and ROS may be involved in gill tissue injury (Kim and Oda, 2010). The toxic role of ROS is generally thought to be indirect or synergistic with other toxins. For instance, in the case of several HAB species, ROS have been shown to stimulate the toxicity of lipid peroxidation products such as polyunsaturated fatty acids (PUFAs) (Arzul *et al.*, 1998; Kim *et al.*, 1999a; Jenkinson and Arzul, 2001; Marshall *et al.*, 2003, 2005b; Mardones *et al.*, 2015). This mode of ROS toxicity helps to explain how transient ROS molecules can exert potentially harmful effects at concentrations that are not directly cytotoxic and over spatio-temporal scales that may exceed ROS lifetimes and diffusive distances. In some cases, cell lysis and the concomitant stimulation of extracellular ROS production are thought to be an important aspect of ichthyotoxicity in fish-killing phytoplankton species (Dorantes-Aranda *et al.*, 2013, 2015; Mardones *et al.*, 2015).

Despite the evidence suggesting that HAB-derived ROS are harmful, chemical additions of ROS that represent concentrations expected during harmful blooms of *H. akashiwo*, *C. marina* and *M. polykrikoides* have been insufficient to completely account for toxic effects on fish and invertebrates (Twiner *et al.*, 2001; Marshall *et al.*, 2003; Woo *et al.*, 2006; Tang and Gobler, 2009a). Such lines of evidence have been used as an argument against the potentially harmful effects of ROS during HABs.

### Other biological interactions

Phytoplankton-derived extracellular ROS may shape other biological interactions, such as grazing, allelopathy, and viral infection. For example, similar lectin-receptor-binding processes have been implicated in the production of extracellular superoxide by phytoplankton (Oda *et al.*, 1998) and the recognition and capture of phytoplankton prey by the microzooplankton species *Oxyrrhis marina* (Wootton *et al.*, 2007). Thus, lectin-stimulated extracellular ROS production by phytoplankton has been proposed to play a role in grazing interactions (Martel, 2009). In fact, extracellular ROS production by *Alexandrium* spp. has been linked to the mortality of microzooplankton grazers (Flores *et al.*, 2012). Extracellular ROS production by *C. marina* and other raphidophytes also modulates interactions with non-predatory organisms, such as the bacterium *Vibrio alginolyticus*, by inhibiting its growth in an antioxidant-dependent manner (Oda *et al.*, 1992b, 1997; Kim *et al.*, 1999b). Furthermore, viral infection of the cosmopolitan phytoplankton species *Emiliania huxleyi* is associated with elevated levels of intracellular ROS and extracellular hydrogen peroxide, although the mechanisms and role(s) of this ROS production are not well understood (Evans *et al.*, 2006).

### Growth

The production of extracellular ROS by a broad diversity of phytoplankton under optimal growth conditions (Fig. 1) suggests that ROS may serve a role in the baseline physiology of these microorganisms. In particular, ROS production may have important consequences for cellular physiology, viability and growth. For example, the removal of superoxide and hydrogen peroxide through the addition of exogenous SOD and catalase, respectively, inhibits the growth of *C. marina* and changes its cell morphology from spindle to round-shaped (Oda *et al.*, 1995). This morphological shift is also observed in *C. antiqua* when superoxide is removed via oxidation by an electrode (Tanaka *et al.*, 1992). These results suggest that extracellular ROS play an essential role in the vitality and survival of *Chattonella* spp. Hansel *et al.* (2016) recently summarized several lines of evidence suggesting a role for extracellular superoxide production in growth regulation by a number of different microbial species. For example, extracellular superoxide is an autocrine growth promoter in other microorganisms such as *Saccharomyces cerevisiae*, *Escherichia coli* and *Salmonella typhimurium*. In these microorganisms, the transition to stationary phase requires a decrease in superoxide concentrations, which is accomplished by cell surface SODs (Saran, 2003; Buetler *et al.*, 2004). Observations demonstrating that biomass-normalized extracellular superoxide production by *C. marina* is highest in exponential phase and lower in stationary phase (Oda *et al.*, 1995; Kawano *et al.*, 1996; Garg *et al.*, 2007) are consistent with the positive relationship between superoxide and growth. Similar growth phase-dependent trends in superoxide production have been observed for other raphidophyte species such as *C. antiqua* and *H. akashiwo* (Skeen *et al.*, 2004; Portune *et al.*, 2010), as well as the dinoflagellate *M. polykrikoides* (Kim *et al.*, 1999a).

Many HAB species modulate cell-normalized ROS production rates in an inverse relationship with cell density (Yang *et al.*, 1995; Twiner and Trick, 2000; Kim *et al.*, 2002; Marshall *et al.*, 2005b), consistent with a potential signaling role for ROS, as recently proposed for the marine cyanobacterium *Trichodesmium* spp. (Hansel *et al.*, 2016). In fact, diluted *C. marina* cultures up-regulate superoxide production rates within timescales of one hour, suggesting that superoxide may act as a dynamic signal to relay information on bloom density (Marshall *et al.*, 2005b), which could potentially be related to growth regulation, as discussed above. ROS signaling does not necessarily imply the production of superoxide by one cell and the detection of that same superoxide anion by another cell, however. Such a process may be unlikely, given the typical lifetime of superoxide in natural waters (Table I). Rather, in the absence of other ROS scavengers, extracellular superoxide produced by one cell may react with cell surface constituents, such as thiols (Winterbourn and Hampton, 2008) and/or lipids (Saran, 2003) on the surface of the same cell, thus generating an endogenous redox signaling cascade.

### Iron acquisition

Besides a potential role as an autocrine growth signal, extracellular ROS production may promote the growth of phytoplankton by more indirect, alternative means via metal nutrient acquisition. For example, superoxide is a potent oxidant and reductant of iron. Under some environmental conditions, extracellular superoxide can increase the bioavailability of iron, especially when this micronutrient is growth-limiting (Rose, 2012). In fact, extracellular superoxide production has been proposed as a strategy for iron acquisition by *Lyngbya majuscula* (Rose *et al.*, 2005), *T. erythraeum* (Roe and Barbeau, 2014), *M. aeruginosa* (Fujii *et al.*, 2011) and *C. marina* (Garg *et al.*, 2007; Liu *et al.*, 2007), although superoxide had no effect on iron uptake by *Thalassiosira* spp. (Kustka *et al.*, 2005) or *Chlorella kessleri* (Middlemiss *et al.*, 2001). Ultimately, the ability of superoxide to facilitate iron acquisition depends on prevailing biogeochemical conditions, which dictate the effect of this ROS on the steady-state concentrations of biologically labile mononuclear inorganic complexes of iron (II) and iron (III) (Rose, 2012). The reader is referred to Rose (2012) for a detailed review on the potential role of extracellular superoxide in microbial iron acquisition.

## FUTURE RESEARCH DIRECTIONS

In aquatic environments, ROS concentrations can be low or undetectable due to rapid reactions with carbon and metals. ROS therefore “invisibly” drive major transformations of key elemental cycles via cryptic biogeochemistry (Hansel *et al.*, 2015). Similarly, we suggest that ROS may play a cryptic role in biological interactions. For example, previous work has revealed that antioxidants can alleviate the toxic effects of ROS-producing HABs (Oda *et al*., 1992b, Yang *et al*., 1995; [Bibr fby039C75][Bibr fby039C78]; Kim *et al.*, 1999a, 1999b; Tang and Gobler, 2009a, 2009b, 2010; Flores *et al.*, 2012), yet representative HAB-derived ROS concentrations are insufficient to induce toxicity (Twiner *et al.*, 2001; Marshall *et al.*, 2003; Woo *et al.*, 2006; Tang and Gobler, 2009a). However, organisms may experience higher doses of ROS than suggested by steady-state ROS concentrations, depending on the underlying kinetics and pathways of ROS production and degradation. For example, ROS concentrations represent the balance of ROS production and decay. Low concentrations of ROS may, therefore, disguise rapid production rates, if decay rates are also high. Depending on the identity and efficiency of ROS-degrading constituents (e.g. PUFAs), high ROS production rates by natural HABs could potentially be toxic without necessarily leading to elevated concentrations of ROS in the surrounding environment.

In order to test this “cryptic interactions” hypothesis, ROS fluxes and concentrations should be assessed together, particularly in natural systems. For example, the majority of phytoplankton-ROS research has been conducted using controlled laboratory experiments with model cultures. Yet much remains to be discovered about phytoplankton-driven ROS dynamics in natural aquatic environments. In fact, the scarcity of ROS measurements during natural HABs makes it difficult to assess whether ROS levels reach toxicity thresholds during these events. In addition to cryptic toxicity, the potential (cryptic) role of phytoplankton-derived extracellular ROS in other biological interactions such as grazing, allelopathy and viral infection should be considered. By potentially mediating biological interactions within and across trophic levels in these ways, ROS may modify food web dynamics and shape aquatic ecosystem health and large-scale biogeochemical cycling via pathways that remain to be discovered.

The inverse dependence of extracellular ROS production rates on phytoplankton cell density and the potential role of extracellular ROS in phytoplankton growth suggest that elevated ROS concentrations and production rates could be expected in aquatic systems leading up to phytoplankton blooms. For instance, in Lake Erie, total hydrogen peroxide concentrations (attributed to biological production) peaked approximately 2 weeks before the appearance of *Microcystis* spp. blooms and the occurrence of maximum microcystin levels during summer 2014 and 2015 (Cory *et al.*, 2016). We suggest that a possible link between phytoplankton bloom formation and elevated biological ROS production could be taxonomically widespread, which has implications for the evaluation and development of ROS-based strategies for predicting algal blooms. In contrast to cryptic cycling, this bloom prediction hypothesis suggests that biological ROS production may peak before a phytoplankton bloom, leading to ROS concentrations and production rates, far from being hidden or invisible, which can be used as a bloom-forecasting index. Given the short lifetimes of ROS (Table I), such a prediction tool could be responsive to ecosystem variables over relatively short timescales, and thus provide a high temporal-resolution indicator (~days to weeks) of potential interest to natural resource managers.

ROS measurements can be technically complex, especially in systems with high organic matter and metal loading. However, future research on phytoplankton-derived ROS is tractable in a range of environments, given currently available geochemical tools for the high sensitivity detection of ROS concentrations and dynamics in complex natural systems (Rose *et al.*, 2008a; Godrant *et al.*, 2009; Heller and Croot, 2010; Yamaguchi *et al.*, 2010; Miller *et al.*, 2011; Burns *et al.*, 2012; Marsico *et al.*, 2015; King *et al.*, 2016; Roe *et al.*, 2016; Zhang *et al.*, 2016b). For a review of ROS quantification methods and technological advancements, the reader is referred to papers by Bartosz (2006), Soh (2006) and Burns *et al.* (2012). In addition to direct ROS measurements, future advances in the understanding of subcellular ROS production mechanisms will lead to new insights on phytoplankton-derived ROS. A variety of methods have been utilized to characterize the molecular basis of ROS production in microorganisms, including gene knockouts (Anderson *et al.*, 2016), as well as chemical activity assays combined with peptide fingerprinting (Andeer *et al.*, 2015) and immunoblotting and immunofluorescence (Kim *et al.*, 2000a). Tracking molecular targets for ROS production in the field could provide critical information on natural ROS dynamics, especially if ROS cycling is rapid and difficult to detect by direct geochemical means.

## Supplementary Material

Supplementary Data
